# New Insights into the Metabolism of Methyltestosterone and Metandienone: Detection of Novel A-Ring Reduced Metabolites

**DOI:** 10.3390/molecules26051354

**Published:** 2021-03-03

**Authors:** Steffen Loke, Lingyu Liu, Maxi Wenzel, Heike Scheffler, Michele Iannone, Xavier de la Torre, Nils Schlörer, Francesco Botrè, Annekathrin Martina Keiler, Matthias Bureik, Maria Kristina Parr

**Affiliations:** 1 Institute of Pharmacy, Pharmaceutical and Medicinal Chemistry, Freie Universität Berlin, Königin-Luise-Straße 2+4, 14195 Berlin, Germany; s.loke@fu-berlin.de (S.L.); lingyu.liu@fu-berlin.de (L.L.); maxi.wenzel@fu-berlin.de (M.W.); heike.scheffler@fu-berlin.de (H.S.); 2 Laboratorio Antidoping FMSI, Largo Giulio Onesti 1, 00197 Rome, Italy; micheleiannone14@gmail.com (M.I.); Xavier.delatorre@fmsi.it (X.d.l.T.); francesco.botre@unil.ch (F.B.); 3 Institute for Organic Chemistry, Universität zu Köln, Grenstraße 4, 50939 Cologne, Germany; nils.schloerer@uni-koeln.de; 4 REDs–Research and Expertise in Antidoping Sciences, ISSUL–Institute del Sciences du Sport de l’Université de Lausanne, 1015 Lausanne, Switzerland; 5 Institute of Doping Analysis & Sports Biochemistry Dresden, Dresdner Str. 12, 01731 Kreischa, Germany; a.keiler@idas-kreischa.de; 6 Environmental Monitoring & Endocrinology, Faculty of Biology, Technische Universität Dresden, Zellescher Weg 20b, 01217 Dresden, Germany; 7 School of Pharmaceutical Science and Technology, Tianjin University, 92 Weijin Road, Nankai District, Tianjin 300072, China; matthias@tju.edu.cn

**Keywords:** 17α-methyl steroids, long-term metabolites, gas chromatography-mass spectrometry, 17-hydroxymethyl-17-methyl-18-nor, D-ring alteration, doping control, metabolism

## Abstract

Metandienone and methyltestosterone are orally active anabolic-androgenic steroids with a 17α-methyl structure that are prohibited in sports but are frequently detected in anti-doping analysis. Following the previously reported detection of long-term metabolites with a 17ξ-hydroxymethyl-17ξ-methyl-18-nor-5ξ-androst-13-en-3ξ-ol structure in the chlorinated metandienone analog dehydrochloromethyltestosterone (“oral turinabol”), in this study we investigated the formation of similar metabolites of metandienone and 17α-methyltestosterone with a rearranged D-ring and a fully reduced A-ring. Using a semi-targeted approach including the synthesis of reference compounds, two diastereomeric substances, viz. 17α-hydroxymethyl-17β-methyl-18-nor-5β-androst-13-en-3α-ol and its 5α-analog, were identified following an administration of methyltestosterone. In post-administration urines of metandienone, only the 5β-metabolite was detected. Additionally, 3α,5β-tetrahydro-epi-methyltestosterone was identified in the urines of both administrations besides the classical metabolites included in the screening procedures. Besides their applicability for anti-doping analysis, the results provide new insights into the metabolism of 17α-methyl steroids with respect to the order of reductions in the A-ring, the participation of different enzymes, and alterations to the D-ring.

## 1. Introduction

**Figure 1:**
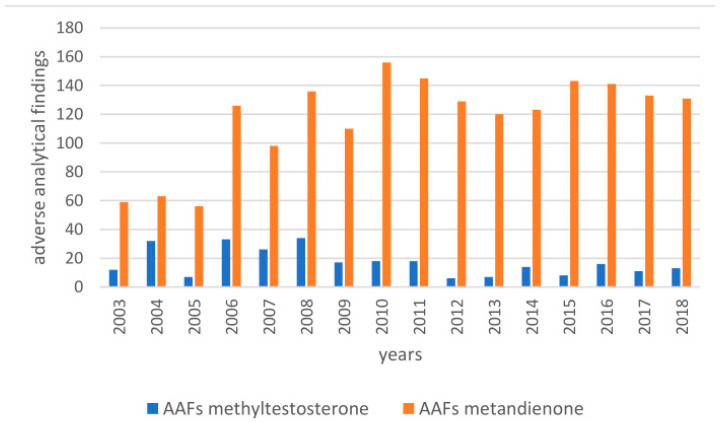
Adverse analytical findings of methyltestosterone and metandienone between 2003 and 2018, according to [4].

Metandienone (17β-hydroxy-17α-methyl-androsta-1,4-dien-3-one, MD, **12**; list of steroids available in supplement S1) and methyltestosterone (17β-hydroxy-17α-methylandrost-4-en-3-one, MT, **18**) are anabolic-androgenic steroids. They were introduced to the market in 1960 (MD) [1] and 1939 (MT) [2] as orally active anabolic androgenic steroids. Although there is no approved drug available anymore, they are still widely marketed and misused as performance-enhancing drugs in sports, even though they are prohibited in and outside of competition by the World Anti-Doping Agency [3]. Many so-called “adverse analytical findings” (AAFs) in doping control have been reported, and their numbers in the last 17 years are displayed in Figure 1 Over the last few years, metandienone and methyltestosterone represent 10% and 1% of all AAFs in the class of anabolic agents. In 2018, only five other substances out of all prohibited compound classes were identified more frequently than metandienone (n = 131), with clenbuterol giving the highest number (n = 320).

Due to the 17α-methyl group, the steroids become orally active, because it prevents the first-pass metabolism by hindering the oxidation of the 17β-hydroxy group sterically, while the introduction of a double bond in position 1 was intended to avoid aromatization and reduced the activity of A-ring-reducing enzymes [5,6,7].

**Figure 2:**
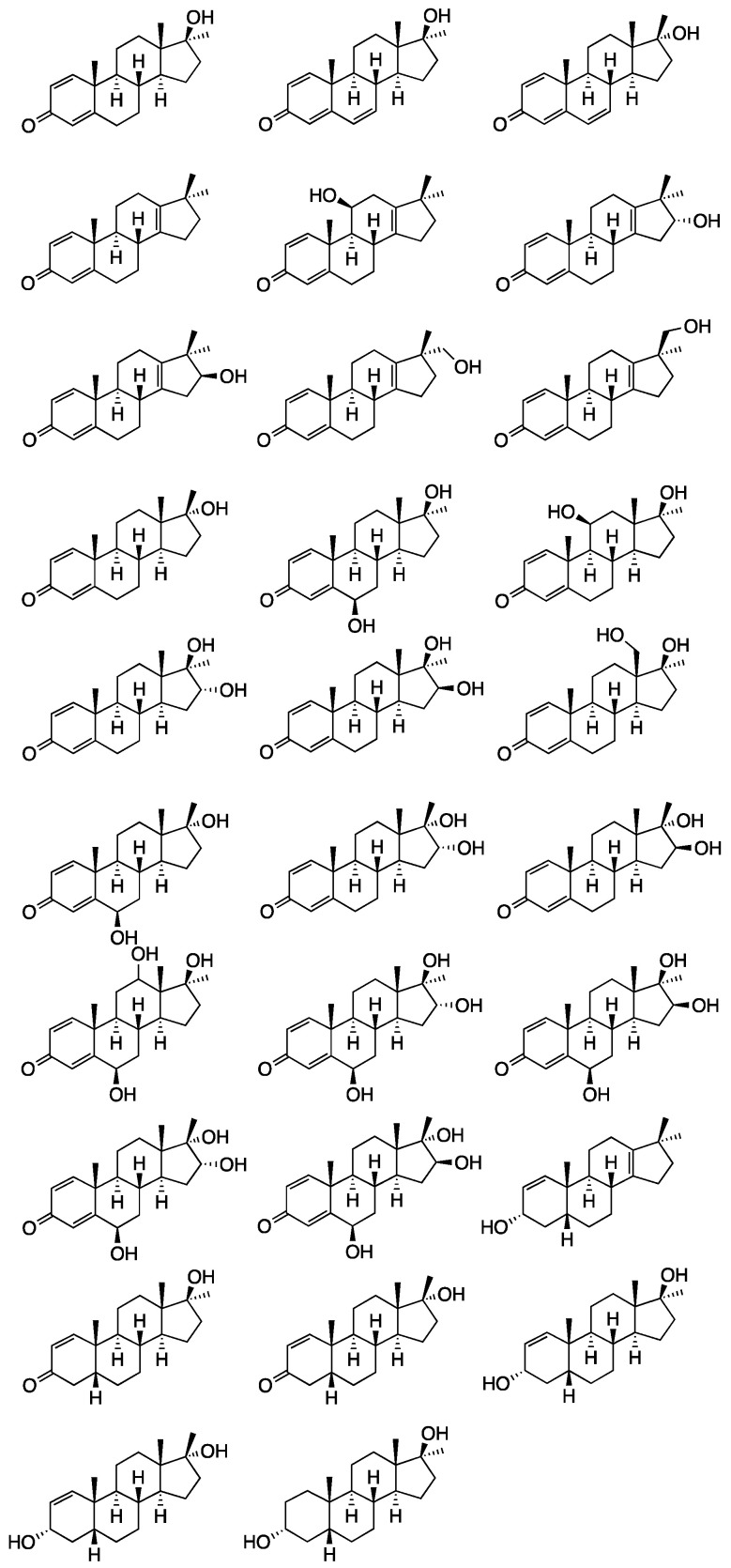
Chemical structures of phase I metabolites of metandienone reported in the literature.

Many metabolites related to the intake of metandienone are reported in the literature, and a number of these have been known for decades. These are generated by both phase I and phase II drug metabolizing enzymes. Phase I reactions include the introduction of a double bond at position 6, the reduction of double bonds in the A-ring, the reduction of the 3-oxo group, hydroxylations in positions 6, 11, 12, 16, or 18, epimerization in position 17, and rearrangement of the D-ring [8,9,10,11,12,13,14,15]. With respect to phase II reactions, both glucuronidation and sulfonation have been reported [16,17]. In recent years, new investigations on long-term metabolites of MD (**12**) identified further metabolites with 17β-hydroxymethyl-17α-methyl-13-ene structure [18,19,20,21]. The known metabolites are shown in Figure 2. Anti-doping laboratories mostly target the parent compound (**12**), 6-OH-metandienone (**13**), epi-metandienone (**14**), epi-metendiol (**15**), nor-epi-metendiol (**16**), and 20βOH-nor-metandienone (**17**) [18,22,23].

**Figure 3:**
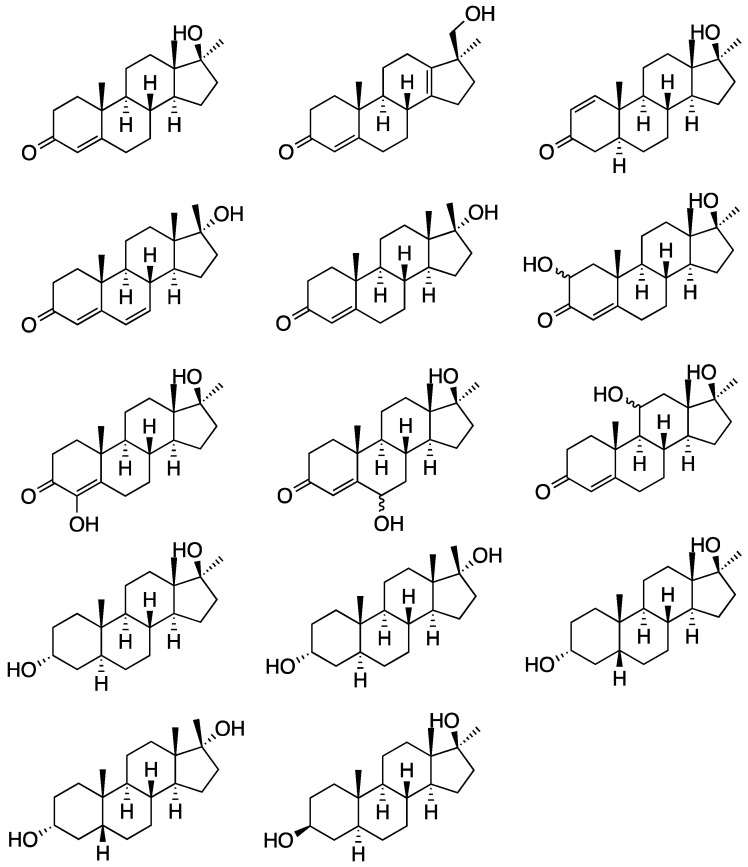
Chemical structures of phase I metabolites of methyltestosterone reported in the literature.

The intake of methyltestosterone leads to several metabolites, which derive from hydroxylations in positions 2, 4, 6, 11, or 20, reduction of the 4,5-double bond, reduction of the 3-oxo group, oxidation yielding a 1,2- or a 6,7-double bond, epimerization in position 17, and rearrangement of the D-ring [24,25,26,27,28]. Subsequent phase II reactions are also leading to both glucuronides and sulfates [26,29]. The structures of metabolites of MT (**18**) are shown in Figure 3. Laboratories mainly screen for the parent compound itself (**18**) and two reduced derivatives (3α5α-THMT, **19**; 3α5β-THMT, **20**). The metabolites of both substances are frequently monitored by gas chromatography-mass spectrometry after hydrolysis of the glycosidic bond of glucuronides as aglycons [22,23].

For other steroids with a similar structure, such as dehydrochloromethyltestosterone, there is a metabolite described with a fully reduced A-ring and a rearranged D-ring [30], which was synthesized in 2018 [31,32]. This metabolite led to an extended detection time of the intake for this substance and thereby increased the number of adverse analytical findings.

As the chemical structures of metandienone (**12**) and methyltestosterone (**18**) are similar to dehydrochloromethyltestosterone, it is conceivable that intake of these substances results in metabolites with a related structure. The discovery of such new metabolites may help in extending the time of detection after the intake of metandienone (**12**) or methyltestosterone (**18**), which would be a considerable contribution to the fight against doping, as cheating may be traced back over a longer period. Additionally, such findings may help to further elucidate the metabolism of synthetic steroids and therefore improve the understanding of human biotransformation.

## 2. Results

### 2.1. Synthesis and Characterization of Reference Steroids

#### 2.1.1. 17-Hydroxymethyl-17-methyl-18-nor-13-enes

**Figure 4:**
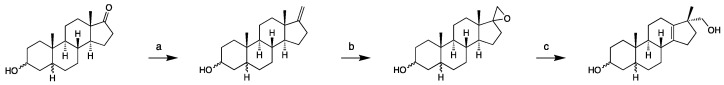
Reaction scheme for 17α-hydroxymethyl-17β-methyl-18-nor-5ξ-androst-13-en-3ξ-ol steroids.

Different diastereomeric 17α-hydroxymethyl-17β-methyl-18-nor-5ξ-androst-13-en-3ξ-ols were synthesized using 3-hydroxyandostan-17-ones as starting material by modifying the D-ring. The method was adapted from Kratena et al. [33] but started with regularly C13β-CH_3_ configured androstanes in contrast to the *ent*-configurated (C13α-CH_3_) androstanes used by Kratena et al. As the first step of synthesis, attachment of an additional carbon-atom at C17 was achieved using Nysted reagent. The epoxidation of the newly introduced 17(20) double bond and subsequent acid catalyzed ring-opening was accompanied by the stereoselective Wagner–Meerwein rearrangement, resulting in 17α-hydroxymethyl-17β-methyl-18-nor-5ξ-androst-13-en-3ξ-ols as the major product, while the 17β-hydroxymethyl-17α-methyl analogs were obtained as minor side products. The reaction scheme is displayed in Figure 4. The preceding synthesis of etiocholanolone (**5**) is described in the supplementary material (S2). The other educt androsterone (**5a**) was obtained from commercial sources.

**Figure 5:**
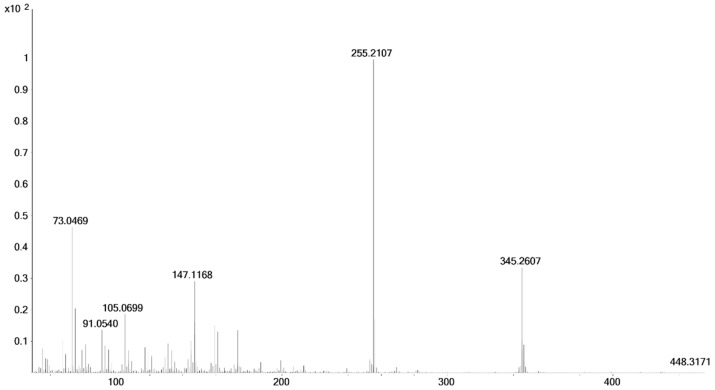
Mass spectrum (GC-EI-QTOF-MS, 70 eV) of 17α-hydroxymethyl-17β-methyl-18-nor-5β-androst-13-en-3α-ol (**8**), bis- trimethylsilyl) (TMS (x-axis: *m*/*z*; y-axis: relative abundance).

As is common in diastereomers, all yielded very similar mass spectra. As an example, the spectrum of 17α-hydroxymethyl-17β-methyl-18-nor-5β-androst-13-en-3α-ol (**8**) is displayed in Figure 5. Using electron ionization at low energy (15 eV, low energy electron ionization, LEI) the molecular ion, which was literally invisible at regular ionization energy (viz. 70 eV), was detected at the accurate mass *m*/*z* 448.3162. The retention time of the bis-trimethylsilyl (TMS) derivatives of the diastereomers are given in Table 1. Further structure confirmation was achieved by 1D and 2D-NMR analysis. Assignments are provided in Table 2.

#### 2.1.2. 17β-Methyl-5β-androstane-3α,17α-diol (**11**)

**Figure 6:**
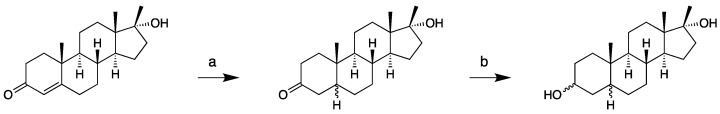
Synthesis route for 17β-methyl-5β-androstane-3α,17α-diol (**11**).

**Figure 7:**
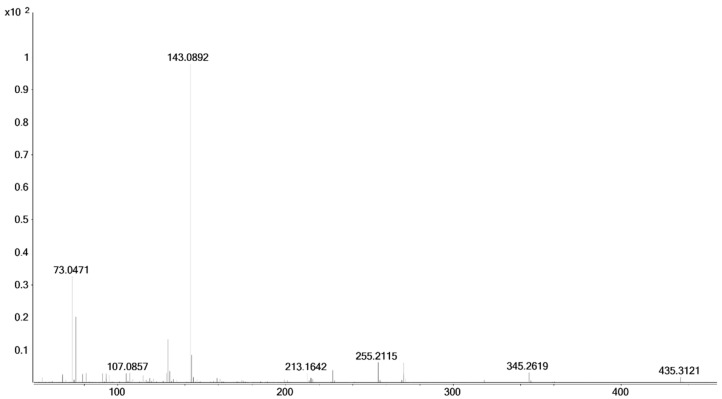
Mass spectrum (GC-EI-QTOF-MS, 70 eV) of 17β-methyl-5β-androstane-3α,17α-diol (**11**), bis TMS (x-axis: *m*/*z*; y-axis: relative abundance).

**Figure 8:**
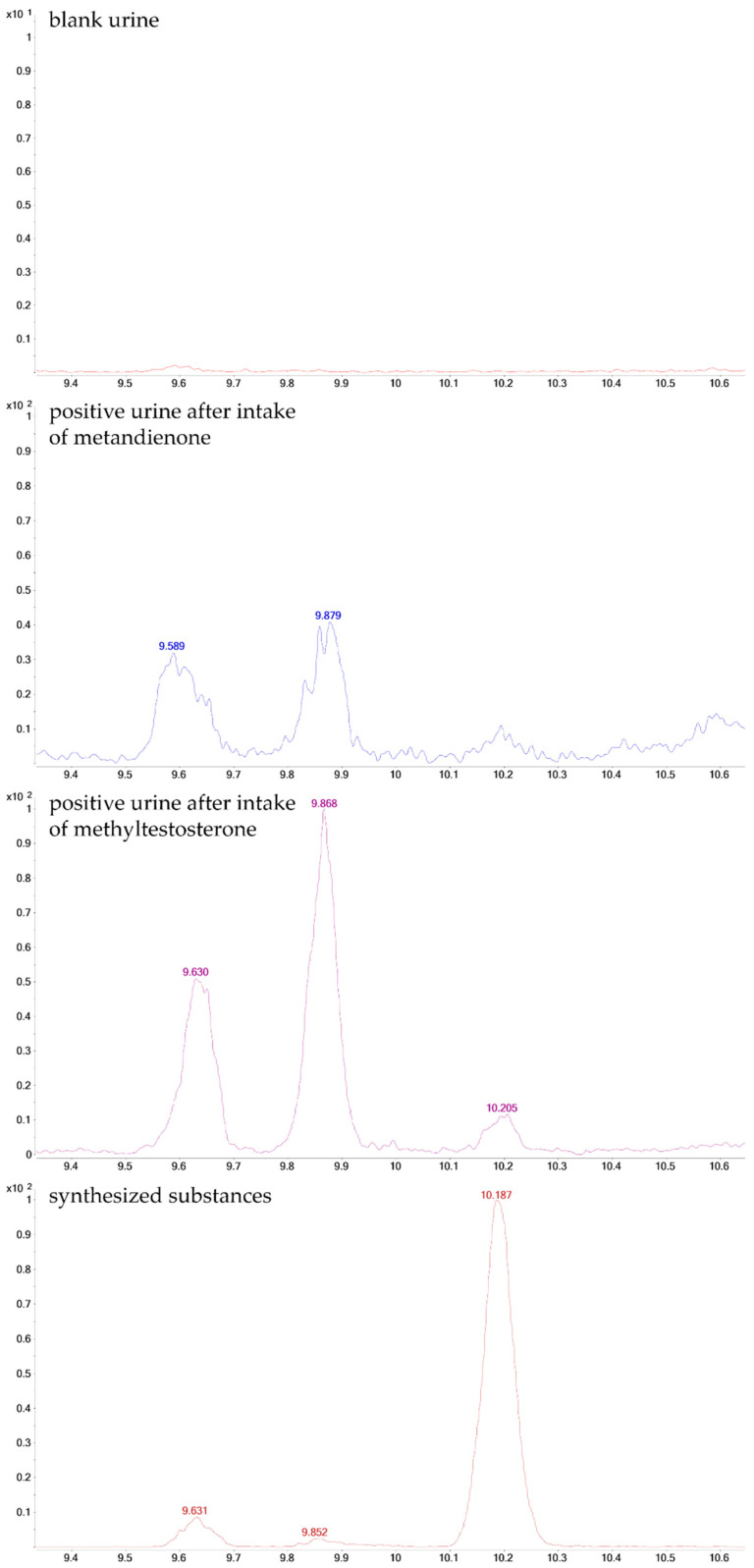
GC-QQQ-MS chromatograms (MRM, *m*/*z* 345 → 173).

Additionally, the diastereomeric 17β-methyl-5ξ-androstane-3ξ,17α-diols were synthesized using epi-methyltestosterone (17α-hydroxy-17β-methyl-androst-4-en-3-one, **9**) as educt. After reduction of the 4,5-double bond and the 3-oxo group, the four fully reduced products **(11**, **11a**, **11b**, **11c)** were obtained as shown in Figure 6. In parallel, reduction of the 3-oxo group in epi-mestanolone (17α-hydroxy-17β-methyl-5α-androstan-3-one, **10a**) yielded the two products **11a** and **11c**. Assignment of the stereochemistry was based on the known stereoselectivity of the reductions, the comparison of the two reactions, and the elution order of the bis-TMS derivatives in GC-MS [24,34]. As a major product 3α,5β-epi-tetrahydromethyltestosterone (**11**) was obtained (Figure 7). The mass spectrum of its bis-TMS derivative is displayed in Figure 8. In LEI the molecular ion was detected at *m*/*z* 450.3352 (accurate mass), confirming the elemental composition C_26_H_50_O_2_Si_2_^+^^•^ (exact mass *m*/*z* 450.3344, difference Δ*m*/*z* = 1.78 ppm).

### 2.2. Post-Administration Urines

Urine samples from the administration trials were analyzed with a GC-QTOF-MS and GC-QQQ-MS after per-TMS derivatization.

The common metabolites of MT (**18**) and MD (**12**) were detected by comparison of retention time and quantifier and qualifier transitions, as reported in Table 3. Corresponding chromatograms are available as supplemental material (S3).

Monitoring of the ion transitions *m*/*z* 345.3 → 255.0, *m*/*z* 345.3 → 173.0, and *m*/*z* 345.3 → 159.0, selected for the 17ξ-hydroxymethyl-17ξ-methyl-18-nor-5ξ-androst-13-en-3ξ-ol isomers, resulted in the detection of two signals at RT_metabolite A_ = 9.56 min and RT_metabolite B_ = 9.80 min in a case of metandienone (**12**). In the post-administration (p.a.) samples of MT (**18**), three signals were detected—one in addition to the two mentioned above (RT_metabolite A_ = 9.56 min, RT_metabolite B_ = 9.80 min, and RT_metabolite C_ = 10.13 min). The comparison with the synthesized reference compounds assigned the metabolites common for MD (**12**) and MT (**18**) to 17α-hydroxymethyl-17β-methyl-18-nor-5β-androst-13-en-3α-ol (**8**) and 3α,5β-epi-tetrahydromethyltestosterone (**11**). The additional metabolite in MT administration was assigned to 17α-hydroxymethyl-17β-methyl-18-nor-5α-androst-13-en-3α-ol (**8a**).

The 3α,5β-epi-tetrahydromethyltestosterone was identified as the first peak in positive urine samples of metandienone and methyltestosterone at 9.56 min (Figure 8).

**Figure 9:**
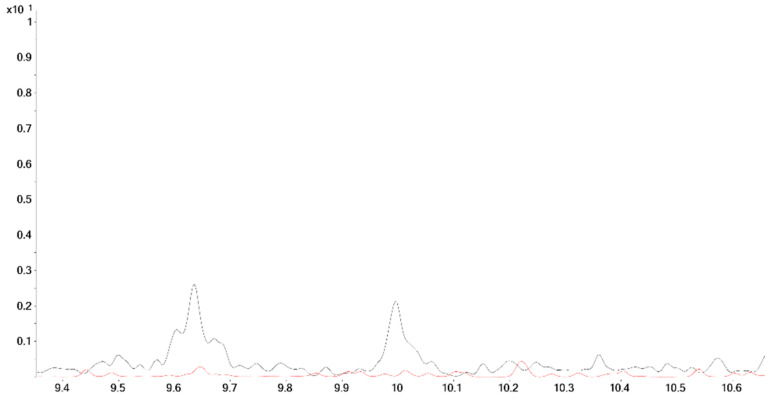
Chromatograms (MRM, *m*/*z* 450 → 345); black: positive urine sample after intake of methyltestosterone; red: synthesized substances.

Another substance with a slightly different structure as compound **11**, namely 3α,5α-epi-tetrahydromethyltestosterone (**11a**), has almost the same retention time as compound **8**. However, **8** does not show the transition *m*/*z* 450 → 345 because of its structure (M^•+^ as TMS-derivative: *m*/*z* 448). As this transition is present in the urine sample, the 3α5α-epi-tetrahydromethyltestosterone (**11a**) can be excluded as the metabolite at 9.80 min (Figure 9). Another closely eluting metandienone metabolite, 17β-methyl-5β-androst-1-ene-3α,17α-diol (15, RT**_15_** = 9.87 min, M^+•^ = 448), was mainly separated and identified by the selective ion transitions given in Table 3.

Two more diastereomers with 17α-methyl-17β-hydroxy configurations (17α-methyl-5α-androstane-3α,17β-diol (**19**) and 17α-methyl-5β-androstane-3α,17β-diol (**20**)) were commercially available and used for retention time comparison and urinary metabolite identification.

## 3. Discussion

### 3.1. Chemical Syntheses and Characterization of Reference Material

The described syntheses starting from etiocholanolone (**5**) or androsterone (**5a**) led to androstane derivatives with a fully reduced A-ring (**8**: 3α-hydroxy-5β-; **8a**: 3-hydroxy-5α‑) and a rearranged D-ring (17α-hydroxymethyl-17β-methyl-18-nor-13-ene). As expected from the reactions, stereochemistry at C3 and C5 was retained unchanged. Due to the commonly known remaining stereochemistry of the 13β-methyl group during the Wagner–Meerwein rearrangement, the 17α-hydroxymethyl-17β-methyl products were the major products as expected. In GC-EI-MS, using common ionization energy of 70 eV, literally no molecular ions were obtained, due to extensive fragmentation. As the dominant fragment, [M-CH_2_-OTMS]^+^ (accurate mass *m*/*z* 345.2607, exact mass *m*/*z* 345.2608, Δ*m*/*z* = −0.29 ppm) was found. The loss of 103 Da is considered characteristic for the TMS derivatized 17α-hydroxymethyl-17β-methyl-18-nor-13-ene steroids [18,35]. The base peak with an accurate mass *m*/*z* 255.2107 (exact mass *m*/*z* 255.2107, Δ*m*/*z* = 0.00 ppm) corresponds to an additional loss of TMSOH. This transition was selected as target in the GC-QQQ-MS method. As qualifiers the transitions to *m*/*z* 159 (C_12_H_15_^+^, accurate mass *m*/*z* 159.1168, exact mass *m*/*z* 159.1168, Δ*m*/*z* = 0.00 ppm) and *m*/*z* 173 (C_13_H_17_^+^, accurate mass *m*/*z* 173.1324, exact mass *m*/*z* 173.1325, Δ*m*/*z* = −0.56 ppm) are monitored.

NMR data confirmed the structure assignments. In 17α-hydroxymethyl-17β-methyl-18-nor-5β-androst-13-en-3α-ol (**8**) stereochemistry at C5 was assigned by the downfield shifted C19 (δ_C19_ = 22.93 ppm) signal. C19 shifts δ_C19_ > 22 ppm are known to be characteristic for 5β-androstanes [36]. Configuration at C3 was deduced from the multiplicity of H3 (δ_H3_ = 3.66 ppm, dddd, *J* = 11/11/5/5 Hz). The diaxial coupling with H-4_ax_ and H-2_ax_ substantiated the axial orientation of H3(β), thus confirming 3α-hydroxy configuration. The NMR data for the residues attached to C17 (δ_C20-CH3_ = 21.72 ppm, δ_H20-CH3_ = 1.00 ppm and δ_C20-CH2OH_ = 68.97 ppm, δ_H20-CH2OH_ = 3.34 ppm and 3.44 ppm) together with NOESY experiments confirmed the 17α-hydroxymethyl-17β-methyl assignment.

In case of 17α-hydroxymethyl-17β-methyl-18-nor-5α-androst-13-en-3α-ol (**8a**), stereochemistry at C5 was assigned by the upfield shifted C19 (δ_C19_ = 10.61 ppm) signal. C19 shifts δ_C19_ < 17 ppm are known to be characteristic in 5α-androstanes [36]. Configuration at C3 was deduced from the multiplicity of H3 (δ_H3_ = 4.08 ppm, dddd, *J* = 3/3/3/3 Hz) representing coupling constants of H-3_eq_ with H-2_eq_, H-2_ax_, H-4_eq_ and H-4_ax_. This substantiated the orientation of H3β, thus confirming 3α-hydroxy configuration. Further confirmation was achieved by selective NOE experiments (irradiation of H19, δ_H19_ = 0.78 ppm). The NMR data for the residues attached to C17 (δ_C20-CH3_ = 21.7 ppm, δ_H20-CH3_ = 0.97 ppm and δ_C20-CH2OH_ = 21.7 ppm, δ_H20-CH2OH_ = 0.97 ppm) together with NOESY experiments confirmed the 17α-hydroxymethyl-17β-methyl assignment.

In comparison to the administered drug, the product of the last synthesis (17β-methyl-5β-androstane-3α,17α-diol, **11**) has a different stereochemistry at C17. Starting from epi-methyltestosterone (17α-hydroxy-17β-methylandrost-4-en-3-one, **9**), the first reduction using hydrogen gas and palladium on charcoal as catalyst leads to the 5β-product (**10**) with huge excess [37]. The subsequent reduction of the 3-oxo group of 5β-dihydro-epi-methyltestosterone (**10**) with sodium borohydride mainly results in the 3α-isomer (88:12, 3α-OH:3β-OH according to Schänzer et al. [24]; **11**). Stereochemistry at C17 is retained during these reactions and thus assigned to 17α-hydroxy-17β-methyl. Due to the collision energy of 70 eV, nearly no molecular ion is found, whereas prominent fragments occur. The two dominant fragments of the above mentioned 17α-hydroxymethyl-17β-methyl steroids, *m*/*z* 345 and *m*/*z* 255, are present in the spectrum of epi-tetrahydromethyltestosterone as well. The signal at *m*/*z* 345 is caused by [M-CH_3_-HOTMS]^+^ (accurate mass *m*/*z* 345.2619, exact mass *m*/*z* 345.2608, Δ*m*/*z* = 3.19 ppm), that at *m*/*z* 255 by another loss of TMSOH (accurate mass *m*/*z* 255.2115, exact mass *m*/*z* 255.2107, Δ*m*/*z* = 3.13 ppm), and the base peak at *m*/*z* 143 by a characteristic D-ring fragment of 17-methyl steroids (accurate mass *m*/*z* 143.0892, exact mass *m*/*z* 143.0887, Δ*m*/*z* = 3.49 ppm).

### 3.2. Urinary Metabolites

As is common in several doping control laboratories, glucuronidated metabolites are enzymatically cleaved and determined as their aglycons together with their analogs that are excreted as unconjugated compounds. Due to the low abundance of some of the target analytes, GC-QQQ-MS in MRM mode is considered as a better-suited technique for metabolite detection after optimization of the ion transitions. As described in the literature [8,24,38], GC-QQQ-MS analysis detected 17α-methyl-5β-androstane-3α,17β-diol (**20**, MT M1) following the administration of both steroids, MD (**12**) and MT (**18**), in all samples. Its 3α,5α-analog (**19**, MT M2) was detected following the administration of MT (**18**), while in MD (**12**) p.a. samples, only very minor corresponding signals were detectable in the 48 h urine and remained unconfirmed due to the low signal-to noise ratio of the qualifier transitions. According to earlier studies, these two metabolites are considered as longest detectable by GC-QQQ-MS after MT (**18**) administration in GC-MS [38]. 

Exclusively after MD (**12**) administration, the parent compound (**12**), epimetendiol (**15**, M1: EMD), 6-hydroxymetandienone (**13**, M2: 6OH-MD), epimetandienone (**14**, M3: EpiMD), normetendiol (17,17-dimethyl-18-nor-5β-androsta-1,13-dien-3α-ol, **16**, M4: NorEMD), and the long-term metabolite 17β-hydroxymethyl-17α-methyl-18-nor-androsta-1,4,13-trien-3-one (**17**, M6: 20βOH-NorMD) were detected, which is in agreement with earlier findings [8,12,13,18]. 

In addition to the commonly monitored metabolites, the two synthesized 17-hydroxymethyl-17-methyl steroids were found in p.a. urines of methyltestosterone (17α-hydroxymethyl-17β-methyl-18-nor-5α-androst-13-en-3α-ol, **8a**, 17α-hydroxymethyl-17β-methyl-18-nor-5β-androst-13-en-3α-ol, **8**) by GC-MS comparison.

Aberrantly, only 17α-hydroxymethyl-17β-methyl-18-nor-5β-androst-13-en-3α-ol (**8**) was confirmed in the p.a. urines of metandienone (**12**). The stereochemistry at C17 is the opposite of the currently monitored long-term metabolite of MD and also to 17β-hydroxymethyl-17α-methyl-18-nor-androsta-4,13-dien-3-one, which was detected earlier after administration of MT [38]. They are also different from the majority of metabolites of analogous 17-methyl steroids [30,31,38,39,40,41,42]. Only less abundant 17α-hydroxymethyl-17β-methyl metabolites of metandienone, methyl-1-testosterone (17β-hydroxy-17α-methyl-5α-androst-1-en-3-one) and oxandrolone [13,41], as well as the recently identified 4-chloro-17α-hydroxymethyl-17β-methyl-18-nor-androsta-4,13-dien-3β-ol (named “M4” by Sobolevsky in 2012) as metabolite of 4-chlorometandienone (dehydrochloromethyltestosterone, active component in Oral Turinabol) [33,43] have a similar stereochemistry at C17.

Interestingly, the structure of the long-term metabolite of 4-chlorometandienone with modified D-ring structure and a fully reduced A-ring (Sobolevsky’s “M3”) was assigned to 4α-chloro-17β-hydroxymethyl-17α-methyl-18-nor-5α-androst-13-en-3α-ol by Forsdahl et al. [31]. The metabolites proposed for MT and MD as described above show an inverse stereochemistry at the D-ring in comparison to these assignments.

Additionally, the product of the last synthesis (17β-methyl-5β-androstane-3α,17α-diol, **11**) has an inverse D-ring at C17 in comparison to the parent compounds and the fully A-ring reduced metabolites, 17α-methyl-5β-androstane-3α,17β-diol (**20**, MT M1) and 17α-methyl-5α-androstane-3α,17β-diol (**19**, MT M2). The latter are formed through reduction of the 1,2- and 4,5-double bond and the 3-oxo group. The 17-epimer was found in the urines after the intake of both mentioned anabolic-androgenic steroids. In the case of MT administration, the metabolite **11** was also described earlier, but found with shorter detection times than the 17α-methyl analogs **19** and **20** [38]. After the intake of MD, this was also found earlier, but with a problem in separation of the four diastereomers [15].

The epimerization of position 17 is a common reaction of 17α-methyl steroids and was first described in 1971 [44]. In humans, it is generated through sulfonation of the tertiary 17-hydroxy group and its subsequent hydrolysis [9]. Besides 17-epimerization, the sulfate may also undergo an elimination of sulfuric acid and concomitant Wagner–Meerwein rearrangement, leading to 17,17-dimethyl-18-norandrosta-1,4,13-trien-3-one. This may undergo A-ring reduction, leading to the metandienone metabolite normetendiol (17,17-dimethyl-18-nor-5β-androsta-1,13-dien-3α-ol, **16**, M4: NorEMD) [11].

Generation of 17α-hydroxymethyl-17β-methyl-18-norandrosta-1,4,13-trien-3-one is generated from the intermediate 17,17-dimethyl-18-norandrosta-1,4,13-trien-3-one by CYP3A4 catalyzed hydroxylation [20], while CYP21A1-catalyzed hydroxylation leads to the formation of a 17β-hydroxymethyl-17α-methyl analog [20].

The stereoselectivity of the A-ring reduction is dependent on the parent compound. For metandienone, there is only very limited generation of metabolites with a 5α-structure. This is is likely due to the 1,2-double bond, which inhibits the activity of 5α-reductase [45]. In contrast, methyltestosterone is metabolized to 5α- and 5β-isomers. This substantiates our hypothesis of metabolite generation due to the A-ring structure with a double bond in position 4 and its already saturated positions 1 and 2 in methyltestosterone, while MD (**12**) has an unsaturated A-ring (i.e., 3-oxo-1,4-diene). Thus, it is reasonable that the 17α-hydroxymethyl-17β-methyl-18-nor-5α-androst-13-en-3α-ol-derivative (**8a**) is only detectable in p.a. samples of methyltestosterone (**18**), while the 5β-analog (**8**) is observed after MT or MD administration. This supports our concept of the order of reductions: if the 1,2-double bond was reduced before the 4,5-double bond, there would have also been 5α-metabolites in p.a. urines of metandienone [8].

**Figure 10:**
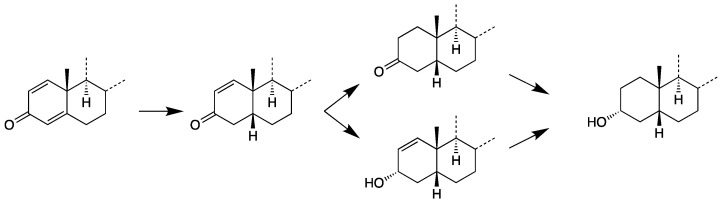
Potential ways of A-ring reduction.

**Figure 11:**
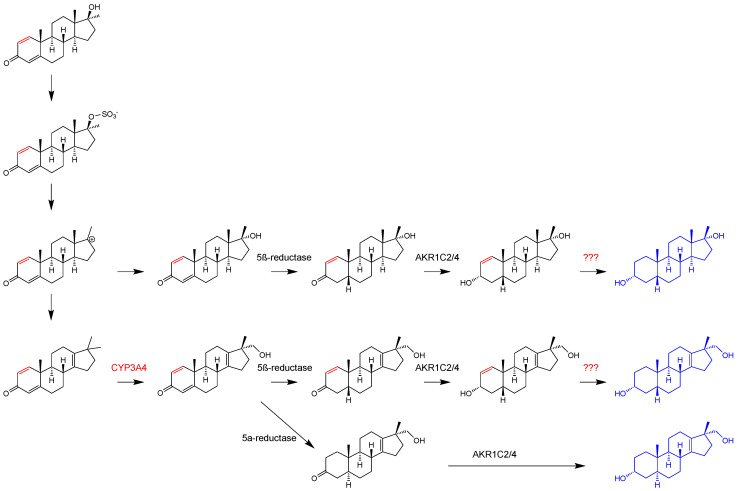
Proposed metabolism of methyltestosterone (black, **18**) and metandienone (red, **12**) to the found metabolites 17α-hydroxymethyl-17β-methyl-18-nor-5β-androst-13-en-3α-ol (**8**), 17α-hydroxymethyl-17β-methyl-18-nor-5α-androst-13-en-3α-ol (**8a**), and 17β-methyl-5β-androstane-3α,17α-diol (**11**) except last step of metandienone. The question marks represent reactions whose enzymes have not been elucidated yet.

Thus, the order of the following two reductions of metandienone (1,2-double bond, 3-oxo group) is not yet confirmed, but it seems to be more likely that the formation of the 3-hydroxy group takes place before the hydrogenation of the 1,2-double bond, because there are known metabolites of metandienone with a 3-hydroxy-1-ene structure but not with a 3-oxo group in a fully reduced A-ring. Both potential ways represent the last step of the proposed formation of the metabolites **8** and **11**. They are displayed in Figure 10. The other reactions of the metabolism of both investigated compounds are displayed in Figure 11.

Based on preliminary data, the mentioned substances are detected for at least 48 h after the intake of parent compounds. Excretion studies with a higher number of volunteers and prolonged sample collection will be performed in the near future to evaluate the detection windows of the new metabolites.

In addition to that, a potential next step will be the investigation of the substrate specificity of 5α-reductase towards 1,2-ene steroids by means of molecular modelling to elucidate structural requirements for generation of 5α-metabolites of androgenic steroids.

The detection and structure identification of the above-mentioned substances in the urine samples help to gain further insights into human metabolism of metandienone and 17α-methyltestosterone. Due to the similarity of other anabolic androgenic steroids to the investigated compounds, it is probable that other metabolites with related structures may be found in further 17α-methyl steroids. Finally, the results may support the fight against doping by introducing new analytes for screening in anti-doping analysis.

## 4. Materials and Methods

### 4.1. Instrumentation

#### 4.1.1. GC-MS/MS

The gas chromatographic-tandem mass spectrometric analysis was performed on an Agilent 7890A gas chromatographic system coupled to an Agilent 7000 GC/MS triple quadrupole mass spectrometer (Agilent Technologies, Milano, Italy). The following conditions for the analysis of the intermediates and products were applied: Agilent HP1 column (17 m, 0.20 mm, 0.11 µm), carrier gas: helium, oven program: 188 °C, hold for 2.5 min, +3 °C/min to 211 °C, hold for 2.0 min, +10 °C/min to 238 °C, +40 °C to 320 °C, hold for 3.2 min, injection volume: 2 µL, split: 20:1, injection temperature: 280 °C, electron ionization (EI): 70 eV, transitions: *m*/*z* 345 → 255 (5 eV), *m*/*z* 345 → 173 (20 eV), *m*/*z* 345 → 159 (10 eV). Prior to injection, samples were treated with 50 µL of trimethyliodosilane (TMIS) reagent (*N*-methyl-*N*-(trimethylsilyl)trifluoroacetamide (MSTFA)/ethanethiol/ammonium iodide, 1000:6:4, *v*:*v*:*w*) at 75 °C for 20 min before analysis to generate the per-TMS derivatives.

#### 4.1.2. GC-QTOF-MS 

High resolution accurate mass analyses were performed on an Agilent GC-QToF 7890B/7250 (Agilent Technologies, Milano, Italy), equipped with an Agilent HP1 column (17 m, 0.20 mm; 0.11 µm) with helium as carrier gas. Injection was performed in split mode with a 1:10 ratio at 280 °C. The oven program had the following heating rates: 188 °C hold for 2.5 min, 3 °C/min to 211 °C and hold for 2 min, 10 °C/min to 238 °C, 40 °C/min to 320 °C, and hold for 3.2 min. The coupled QToF was operated in full scan with an ionization energy of 70 eV. Aberrantly, in LEI an ionization energy of 15 eV was applied. Ions were detected from *m*/*z* 50 to 750.

#### 4.1.3. HPLC Purification

The purification of the synthesized reference steroids was performed by semi-preparative HPLC using an Agilent 1260 Infinity Quaternary HPLC system coupled to an Agilent Infinity 1260 diode array detector (Agilent Technologies GmbH, Waldbronn, Germany). Chromatographic separation was achieved on a Hypersil ODS C18 column (pore size: 120 Å, 250 mm length, 10 mm ID, 5 μm particle size, Thermo Scientific, Schwerte, Germany). Isocratic elution was accomplished at a flow rate of 3 mL/min using acetonitrile:water (7:3, *v*:*v*) as the mobile phase. The UV signal was monitored at 194 nm.

#### 4.1.4. Nuclear Magnetic Resonance

The nuclear magnetic resonance (NMR) analyses were performed at 500 MHz (^1^H NMR) and 125 MHz (^13^C NMR) at 296 K on a Bruker (Rheinstetten, Germany) Avance III instrument equipped with a nitrogen-cooled 5 mm inverse TCI cryoprobe with actively shielded z-gradient coil. Chemical shifts are reported in δ values (ppm) relative to tetramethylsilane. Solutions of about 5 mg of each compound in deuterated dimethylsulfoxide (*d*_6_-DMSO) were used for conducting ^1^H; H,H COSY; ^13^C; edited HSQC; HMBC, selective NOE and NOESY experiments. Two-dimensional experiments were recorded in non-uniform sampling (NUS) mode.

### 4.2. Chemicals and Reagents

Androst-4-ene-3,17-dione (**1**) was purchased from TCI (Tokyo, Japan), androsterone (**5a**), and TiCl_4_ from Acros Organics (Fair Lawn, New Jersey, USA), 17α-methyltestosterone (**18**), palladium on charcoal, nysted reagent, K-Selectride and meta-chloroperoxybenzoic acid from Aldrich (Steinheim, Germany). 17β-Methyltestosterone (epi-MT, **9**) was obtained from Santa Cruz Biotechnology (Heidelberg, Germany). 17α-methyl-5β-androstane-3α,17β-diol (**20**) and 17α-methyl-5α-androstane-3α,17β-diol (**19**) were purchased from the National Measurement Institute (North Ryde, Australia). Benzene was delivered from Thermo Fisher (Karlsruhe, Germany), hexane, ethyl acetate, methanol, dichloromethane, diethyl ether, hydrochloric acid, sodium bicarbonate, and potassium carbonate from Fisher Scientific (Loughborough, United Kingdom). MSTFA was obtained from from Chemische Fabrik Karl Bucher GmbH (Waldstetten, Germany). THF, monosodium phosphate, sodium borohydride, and p-toluenesulfonic acid were purchased from Merck (Darmstadt, Germany), while TBME, potassium hydroxide, and sodium carbonate were bought from Carl Roth (Darmstadt, Germany). Hydrogen gas was provided by Air Liquide (Düsseldorf, Germany) and β-glucuronidase from Roche Diagnostics (Mannheim, Germany). All other chemicals were purchased from VWR (Darmstadt, Germany).

### 4.3. Synthesis of Reference Steroids

#### 4.3.1. Diastereomeric 17-hydroxymethyl-17-methyl-18-nor-5-androst-13-en-3-ols

17-Methylene-5ξ-androstan-3ξ-ol (**6**, **6a**)

A flask was flushed with argon gas and held under an argon atmosphere. After cooling to 0 °C, Nysted reagent (20%) was diluted with absolute tetrahydrofurane (THF abs.), and titanium tetrachloride was added dropwise. After 15 min of stirring, the mixture was brought to room temperature. The precursor steroid (**5**, or **5a**) was dissolved in 10 mL THF (abs.) and was added dropwise to the mixture. The reaction was held under these conditions overnight. After cooling down the mixture to 0 °C, aqueous hydrochloric acid (2 M) and ice-cold water were added, and it was extracted four times with diethyl ether. The organic phases were combined, washed with sodium hydrogen carbonate and brine, dried over sodium sulfate, and evaporated to dryness. Detailed amounts of reactants and solvents are available in the supplements (S4).

Spiro[5 ξ-androstan-17,2′-oxirane]-3ξ-ol (**7**, **7a**)

The crude substance was dissolved in dichloromethane, and potassium hydrogen carbonate and meta-chloroperoxybenzoic acid were added. The solution was stirred for 3 h at ambient temperature. Afterwards, the mixture was poured into water and extracted three times with dichloromethane. The organic phases were washed with brine and then dried over sodium sulfate. Further details are disclosed as supplemental information.

17α-Hydroxymethyl-17β-methyl-18-nor-5β-androst-13-en-3α-ol (**8**)

The intermediate product **7** was dissolved in 5 mL methanol plus 5 mL aqueous hydrochloric acid (1 M). The solution was stirred overnight. Then, 10 mL water were added, and the mixture was extracted three times with 20 mL t-butyl methyl ether. The organic phases were combined and dried over sodium sulfate. The product was purified by column chromatography (silica gel 60, 300 mm × 30 mm, particle size 40–63 µm), using hexane/ethyl acetate (3:2, *v*:*v*) followed by HPLC fractionation. The finally purified product (**8**) was obtained in a total amount of 16 mg (yield: 1.51%, purity >98%).

17α-Hydroxymethyl-17β-methyl-18-nor-5α-androst-13-en-3α-ol (**8a**)

The intermediate product **7a** was dissolved in 20 mL of methanol and 20 mL of aqueous hydrochloric acid (1 M). The solution was stirred overnight. Then, 30 mL of water was added, and the mixture was extracted three times with 50 mL t-butyl methyl ether. The organic phases were combined and dried over sodium sulfate. The product was purified by column chromatography (silica gel 60, 300 mm × 30 mm, particle size 40–63 µm), using hexane/ethyl acetate (3:2, *v*:*v*) followed by HPLC fractionation. A total amount of 263 mg (yield: 57.1%, purity >98%) of the final product (**8a**) was obtained.

#### 4.3.2. Epi-Tetrahydromethyltestosterones

17β-Μethyl-5β-androstane-3α,17α-diol (**11**)

A mixture of 450 µL methanol and 50 µL potassium hydroxide solution (5 M) was prepared, and 100 µg epi-methyltestosterone (**9**) was dissolved. A spatula tip of palladium on charcoal was added, and hydrogen gas flushed through the solution for 5 min. After adding 2 mL of water, the mixture was extracted three times with 3 mL of hexane and evaporated to give the product **10**. The residue was dissolved in methanol/water (9:1, *v*:*v*) and a spatula tip of sodium borohydride was added. The solution was stirred for one hour at room temperature. After adding ammonium chloride to stop the reaction, potassium hydroxide solution (1 M) was added to yield alkaline solution. Then, the solution was extracted three times with dichloromethane and evaporated to give the product **11**.

17β-Μethyl-5α-androstane-3α,17α-diol (**11a**)

A spatula tip of epi-mestanolon (**10a**) was dissolved in 2 mL of absolute THF, 80 µL of K-Selectride was added and the mixture was stirred for 1 h at ambient temperature. Afterwards, 100 µL of aqueous hydrochloric acid (1 M) was added until there was no formation of bubbles anymore. Then, 150 µL of potassium hydroxide solution (1 M) was added and the mixture was extracted three times with 5 mL of hexane. The hexane-phase was evaporated to give the product **11a**.

### 4.4. Human Administration Trial

Urine samples out of the stock of the anti-doping laboratory in Rome were available for analysis. Samples collected before and after an oral administration of either MD or MT were used for evaluation of the excretion of the hypothized metabolites. The excretion study with MT was carried out by a healthy male volunteer (Caucasian, 50 years old, 80 kg and normal body mass index). A single oral dose of 10 mg of MT (Metadren^®^, Novartis, Basel, Switzerland) was administered. For investigation of MD metabolism, a single oral dose of 5 mg MD (Dianabol^®^, Ciba-Geigy, Basel, Switzerland) was administered to a healthy male volunteer (Caucasian, 45 years old, 82 kg and normal body mass index).

### 4.5. Urine Sample Preparation

An aliquot of 6 mL urine was used for the following analysis. As internal standard methyltestosterone (50 µL of a solution of 100 µg/mL) was added. After the addition of 750 µL of phosphate buffer (0.8 M) and 50 µL β-glucuronidase, the mixture was incubated at 55 °C for 60 min. Afterwards, 500 µL of carbonate buffer (20%) was added and the mixture was extracted with 10 mL of TBME. After evaporation, 50 µL of TMIS reagent was added to the sample and the mixture was treated at 75 °C for 20 min before analysis to generate the per-TMS derivatives.

## Figures and Tables

**Table 1:** Retention times (GC-QQQ-MS), molecular ions (M^•+^) in low electron ionization (LEI, 20 eV), and mass difference to exact mass (*m*/*z*_ther_ 448.3187, C_26_H_48_O_2_Si_2_^+•^) of diasteromeric 17α-hydroxymethyl-17β-methyl-18-nor-5ξ-androst-13-en-3ξ-ols as per-TMS derivatives.

No.	Stereochemical Assignment	RT [min]	Molecular Ion (LEI)	Δ*m*/*z* [ppm]
**8**	3α, 5β, 17α-CH_2_OH	9.80	448.3162	−5.6
**8a**	3α, 5α, 17α-CH_2_OH	10.13	448.3164	−5.1

**Table 2:** ^1^H and ^13^C NMR spectral data of 17α-hydroxymethyl-17β-methyl-18-nor-5β-androst-13-en-3α-ol (**8**) and 17α-hydroxymethyl-17β-methyl-18-nor-5α-androst-13-en-3α-ol (**8a**). Multiplicity of signals indicated as singlet (s), doublet (d).

	17α-hydroxymethyl-17β-methyl-18-nor-5β-androst-13-en-3α-ol (8)		17α-hydroxymethyl-17β-methyl-18-nor-5α-androst-13-en-3α-ol (8a)	
	δ_C_	δ_H_	δ_C_	δ_H_
1	35.18	α: 1.92 β: 1.06	31.93	α: 1.35 β: 1.58
2	30.64	α: 1.37 β: 1.72	28.93	α: 1.66 β: 1.75
3	71.75	β: 3.66	66.43	β: 4.08
4	36.59	α: 1.75 β: 1.56	35.70	α: 1.41 β: 1.53
5	41.75	β: 1.47	39.03	α: 1.60
6	27.63	α: 1.35 β: 1.93	28.86	α: 1.25 β: 1.30
7	26.08	α: 1.72 β: 1.21	31.47	α: 1.04 β: 1.95
8	37.41	β: 2.14	36.97	β: 2.10
9	38.44	α: 1.67	52.03	α: 1.01
10	34.67	-	36.13	-
11	22.43	α: 1.79 β: 1.14	22.14	α: 1.90 β: 1.16
12	22.65	α: 1.83 β: 2.02	22.59	α: 1.80 β: 2.01
13	135.94	-	135.85	-
14	141.76	-	141.81	-
15	30.60	α: 2.32 β: 2.12	30.61	α: 2.33 β: 2.11
16	34.20	α: 1.58 β: 1.97	34.13	α: 1.97 β: 1.58
17	51.66	-	51.54	-
19	22.93	0.93 (s)	10.61	0.78 (s)
20βCH_3_	21.72	1.00 (s)	21.75	0.99 (s)
20αCH_2_OH	68.97	3.34 (d) 3.44 (d)	68.99	3.31 (d) 3.42 (d)

**Table 3:** Retention times (GC-QQQ-MS) and ion transitions of currently targeted metabolites in anti-doping analysis.

Compound (Parent Compound)	RT [min]	Ion Transitions (*m*/*z*) & Collision Energies
17β-methyl-5β-androst-1-ene-3α,17α-diol (**15**)	9.87	358.0 → 301.0 (10 eV) 358.0 → 169.0 (30 eV) 358.0 → 196.0 (10 eV) 358.0 → 194.0 (10 eV) 216.0 → 159.0 (5 eV) 268.0 → 211.0 (10 eV) 216.0 → 187.0 (5 eV)
6β,17β-dihydroxy-17α-methyl-androsta-1,4-dien-3-one (**13**)	16.19	517.5 → 229.0 (5 eV) 517.5 → 297.0 (5 eV) 517.5 → 205.0 (30 eV) 517.5 → 429.4 (5 eV)
17α-hydroxy-17β-methyl-androsta-1,4-dien-3-one (**14**)	13.77	444.4 → 206.0 (10 eV) 444.4 → 191.0 (30 eV) 339.0 → 270.0 (20 eV) 444.4 → 283.0 (30 eV)
17,17-dimethyl-18-nor-5β-androsta-1,13-dien-3α-ol (**16**)	6.19	253.0 → 185.0 (20 eV) 253.0 → 197.0 (20 eV) 253.0 → 105.0 (30 eV) 216.0 → 131.0 (20 eV) 216.0 → 145.0 (20 eV)
17β-hydroxymethyl-17α-methyl-18-nor-androsta-1,4,13-trien-3-one (**17**)	13.84	236.0 → 133.0 (5 eV) 339.0 → 193.0 (20 eV) 442.4 → 243.0 (15 eV) 442.4 → 133.0 (15 eV) 339.0 → 133.0 (20 eV) 339.0 → 243.0 (20 eV)
17α-methyl-5β-androstane-3α,17β-diol (**20**)	13.36	228.0 → 174.0 (5 eV) 270.0 → 157.0 (30 eV) 270.0 → 171.0 (30 eV) 270.0 → 199.0 (30 eV)
17α-methyl-5α-androstane-3α,17β-diol (**19**)	13.22	318.0 → 199.0 (10 eV) 318.0 → 187.0 (10 eV) 318.0 → 182.0 (10 eV) 450.4 → 365.0 (10 eV) 450.4 → 261.0 (10 eV)

## Data Availability

Raw data are stored at the authors.

## Supplementary Materials

The following are available online: Supplement S1: Table of steroids, Supplement S2: Preceding synthesis of etiocholanolone, Supplement S3: Chromatograms of urine samples. Supplement S4: Detailed amounts of reactants and solvents in the synthesis of 17-hydroxymethyl-17-methyl-18-nor-5-androst-13-en-3-ols.

## Sample Availability

Samples of the compounds are kept amongst the authors.
