# Supplementary material for: New Insights into the Metabolism of Methyltestosterone and Metandienone: Detection of Novel A-Ring Reduced Metabolites
Source: Molecules. 2021 Mar 3;26(5):1354. doi: 10.3390/molecules26051354 (PMC7961831; doi:10.3390/molecules26051354)
Supplement: Supplementary file 1 [file molecules-26-01354-s001.zip › molecules-1132802-final-SM/210202_Molecules_20OHTHMT_Supplement 1.pdf]

molecules

MDPI

Supplement S1. List of steroids

No.	Systematic Name	Structure
1	Androst-4-ene-3,17-dione	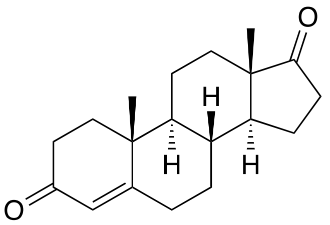
2	Spiro[androst-4-ene-17-2′-[1,3]-dioxolan]-3-one	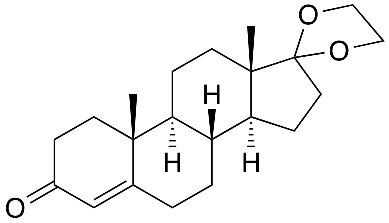
3	Spiro[5β-androstane-17-2′-[1,3]-dioxolan]-3-one	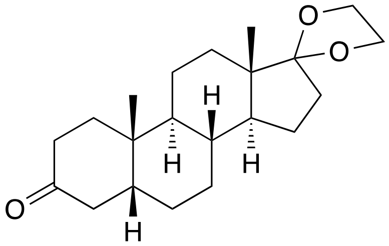
4	Spiro[5β-androstane-17-2′-[1,3]-dioxolan]-3α-ol	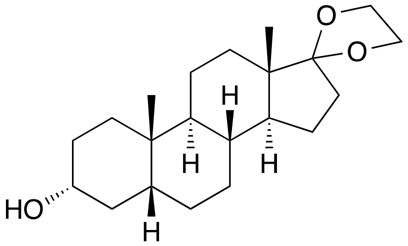
5	3α-Hydroxy-5β-androstan-17-one	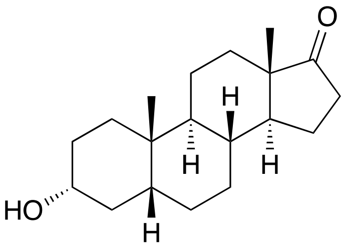

5a	3α-Hydroxy-5α-androstan-17-one	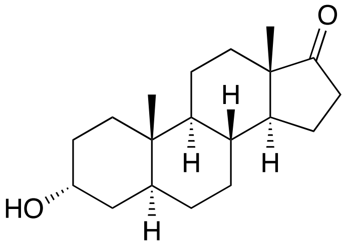
6	17-Methylen-5β-androstan-3α-ol	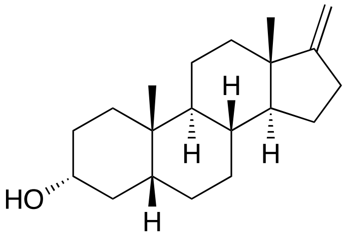
6a	17-Methylen-5α-androstan-3α-ol	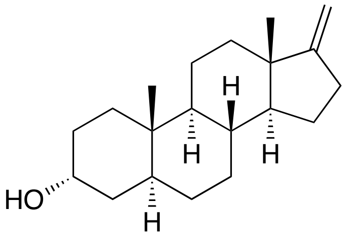
7	Spiro[5β-androstan-17,2′-oxiran]-3α-ol	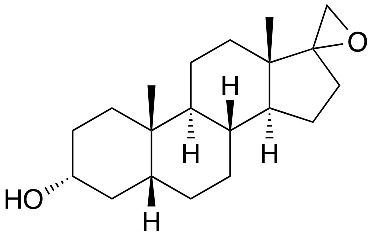
7a	Spiro[5α-androstan-17,2′-oxiran]-3α-ol	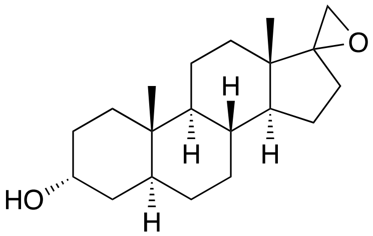
8	17α-Hydroxymethyl-17β-methyl-18-nor-5β-androst-13-en-3α-ol	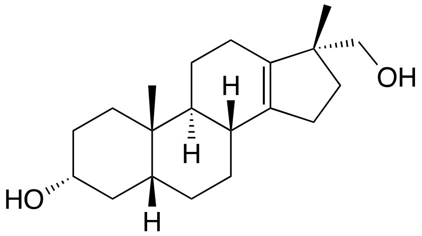
8a	17α-Hydroxymethyl-17β-methyl-18-nor-5α-androst-13-en-3α-ol	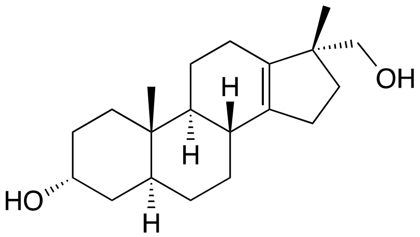

9	17*α*-Hydroxy-17β-methyl-androst-4-en-3-one	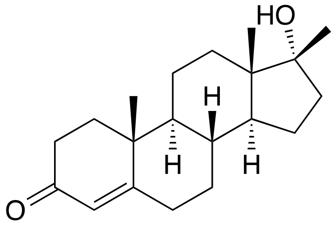
10	17*α*-Hydroxy-17β-methyl-5β-androstan-3-one	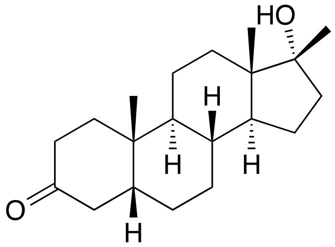
10a	17*α*-Hydroxy-17β-methyl-5*α*-androstan-3-one	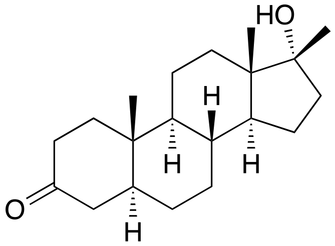
11	17β-Methyl-5β-androstane-3*α*,17*α*-diol	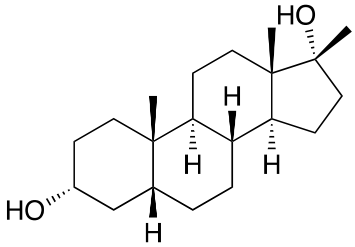
11a	17β-Methyl-5*α*-androstane-3*α*,17*α*-diol	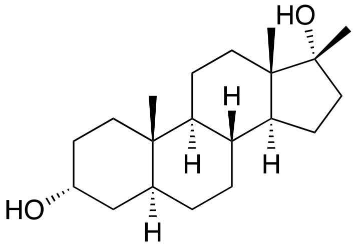
11b	17β-Methyl-5β-androstane-3β,17*α*-diol	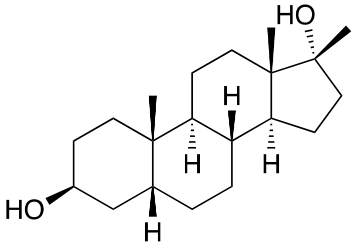
11c	17β-Methyl-5*α*-androstane-3β,17*α*-diol	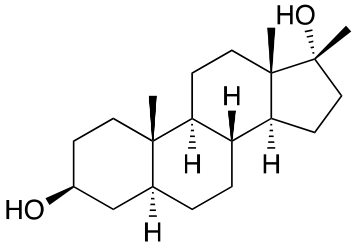

12	17β-Hydroxy-17α-methyl-androsta-1,4-dien-3-one	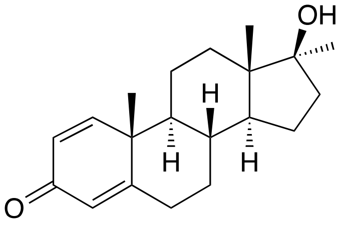
13	6β,17β-Dihydroxy-17α-methyl-androsta-1,4-dien-3-one	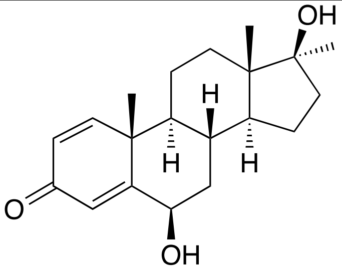
14	17α-Hydroxy-17β-methyl-androsta-1,4-dien-3-one	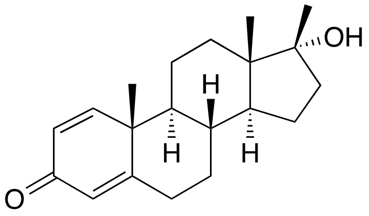
15	17β-Methyl-5β-androst-1-en-3α,17α-diol	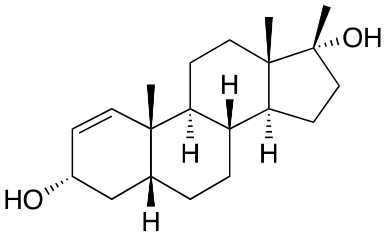
16	17,17-Dimethyl-18-nor-5β-androsta-1,13-dien-3α-ol	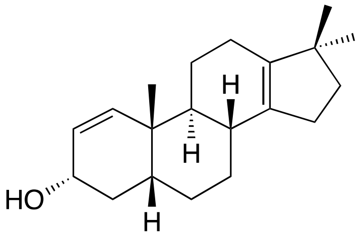
17	17β-Hydroxymethyl-17α-methyl-18-nor-androsta-1,4,13-trien-3-one	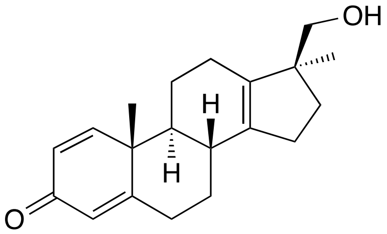
18	17β-Hydroxy-17α-methyl-androst-4-en-3-one	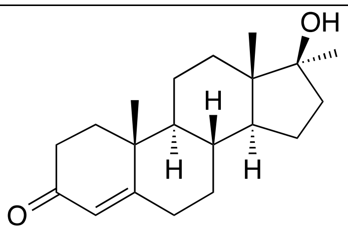

19	17α-Methyl-5α-androstane-3α,17β-diol	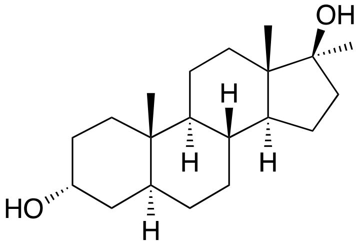
20	17α-Methyl-5β-androstane-3α,17β-diol	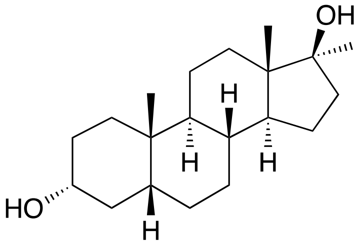
