# Supplementary material for: New Insights into the Metabolism of Methyltestosterone and Metandienone: Detection of Novel A-Ring Reduced Metabolites
Source: Molecules. 2021 Mar 3;26(5):1354. doi: 10.3390/molecules26051354 (PMC7961831; doi:10.3390/molecules26051354)
Supplement: Supplementary file 1 [file molecules-26-01354-s001.zip › molecules-1132802-final-SM/210202_Molecules_20OHTHMT_Supplement2.pdf]

molecules

MDPI

Supplement S2. Preceding synthesis of etiocholanolone

*S2.1. Spiro[androst-4-ene-17-2′-[1,3]-dioxolan]-3-one (**2**)*

To a solution of androstenedione **1** (1 g, 3.5 mmol) in 30 mL benzene, 235 µL ethylene glycol and 39 mg p-toluenesulfonic acid monohydrate were added. The solution was boiled under reflux for 100 minutes. The mixture was neutralized with a sodium carbonate solution (10 %) and dried over sodium sulfate. The benzene was evaporated. Afterward, the crude product was recrystallized in n-hexane / ethyl acetate (15 + 2).

*S2.2. Spiro[5β-androstane-17-2′-[1,3]-dioxolan]-3-one (**3**)*

The acetal **2** (720 mg) was dissolved in 30 mL of a mixture of methanol and potassium hydroxide solution (5 M) (9 + 1), and 360 mg palladium on charcoal was added. The solution was first flushed and then held under hydrogen gas (4 bar) for 24 hours. The product was separated by filtration through celite and dried over phosphorus pentoxide.

*S2.3. Spiro[5β-androstane-17-2′-[1,3]-dioxolan]-3α-ol (**4**)*

The androstane derivative **3** was dissolved in 50 mL of a mixture of methanol and water (9 + 1), and 33 mg sodium borohydride was added. The solution was stirred for 60 minutes at room temperature. Afterward, hydrochloric acid (1 M) was added until gas formation stopped. The organic layer was reduced by evaporation, then brought to pH 12, and finally extracted three times with 50 mL dichloromethane. The organic phases were combined and evaporated to dryness.

*S2.4. 3α-Hydroxy-5β-androstan-17-one (**5**)*

The substance **4** was dissolved in 100 mL acetic acid (30 %) and boiled under reflux for 45 minutes. Then, 70 mL of water was added, and it was extracted three times with 100 mL dichloromethane. The organic phases were combined and evaporated to dryness. Afterward, the crude product was purified by column chromatography (silica gel, hexane + ethyl acetate 3 + 2; silica gel, hexane + ethyl acetate 7 + 3) to give 43 mg of etiocholanolone **5**.
