# Supplementary material for: New Insights into the Metabolism of Methyltestosterone and Metandienone: Detection of Novel A-Ring Reduced Metabolites
Source: Molecules. 2021 Mar 3;26(5):1354. doi: 10.3390/molecules26051354 (PMC7961831; doi:10.3390/molecules26051354)
Supplement: Supplementary file 1 [file molecules-26-01354-s001.zip › molecules-1132802-final-SM/210202_Molecules_20OHTHMT_Supplement3.pdf]

molecules

MDPI

Supplement S3: Chromatograms of urine samples

Post administration urine of metandienone, collected 48 h after ingestion

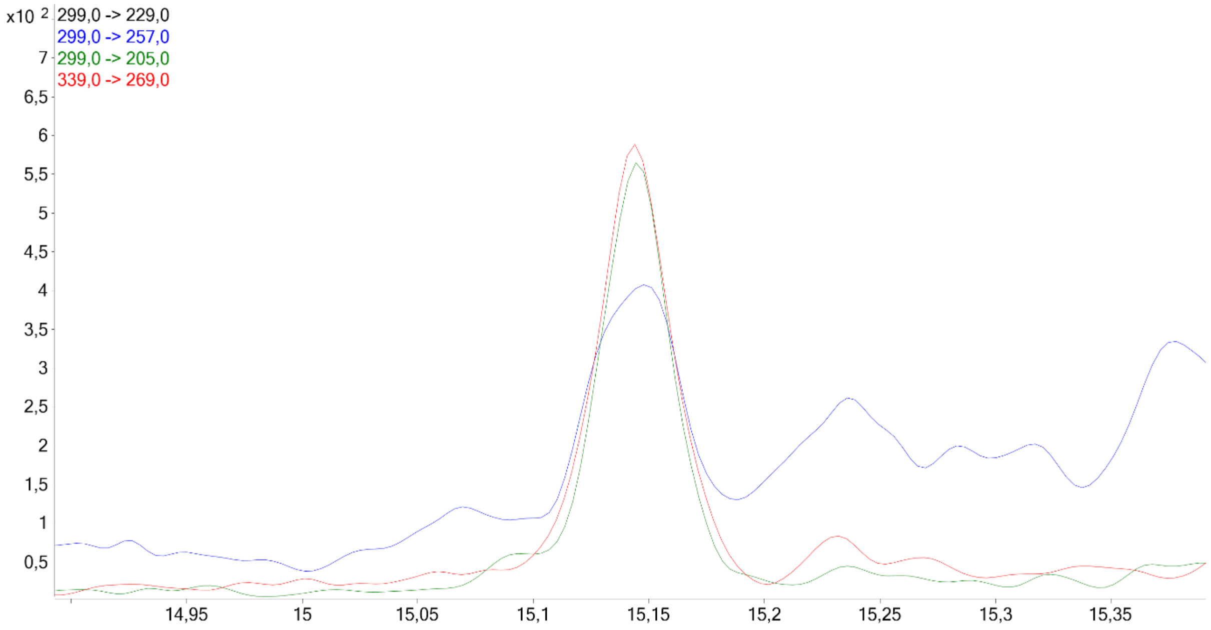

Figure S3-1: GC-QQQ-MS chromatogram of metandienone (**12**)

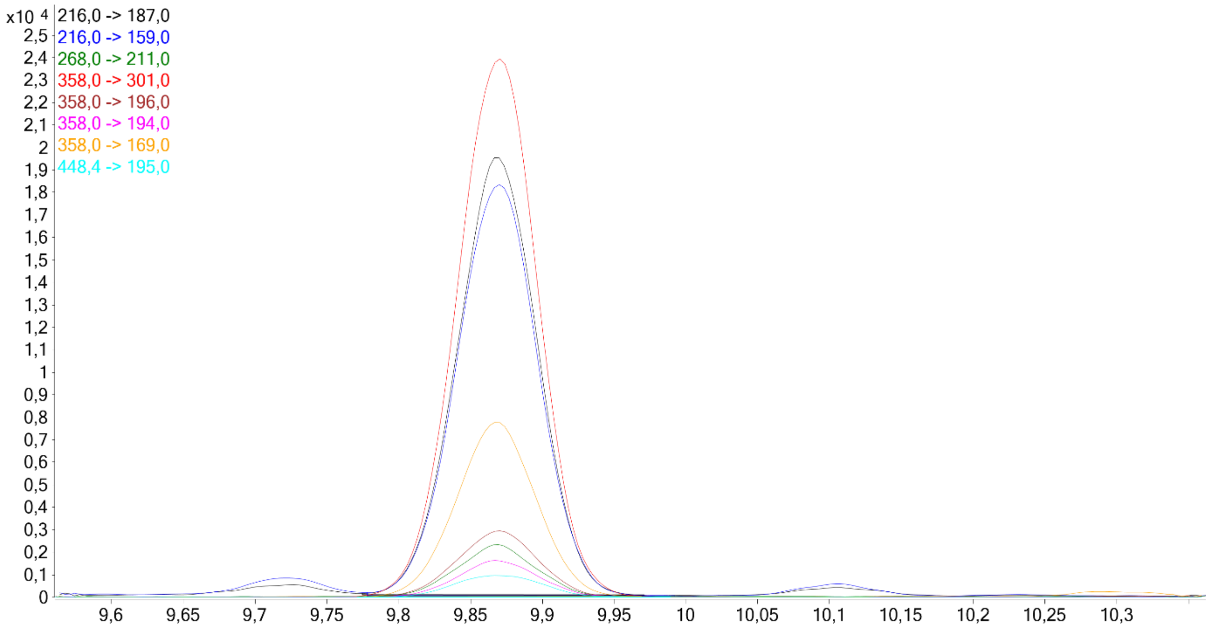

Figure S3-2: GC-QQQ-MS chromatogram of epimetendiol (M1, **15**)

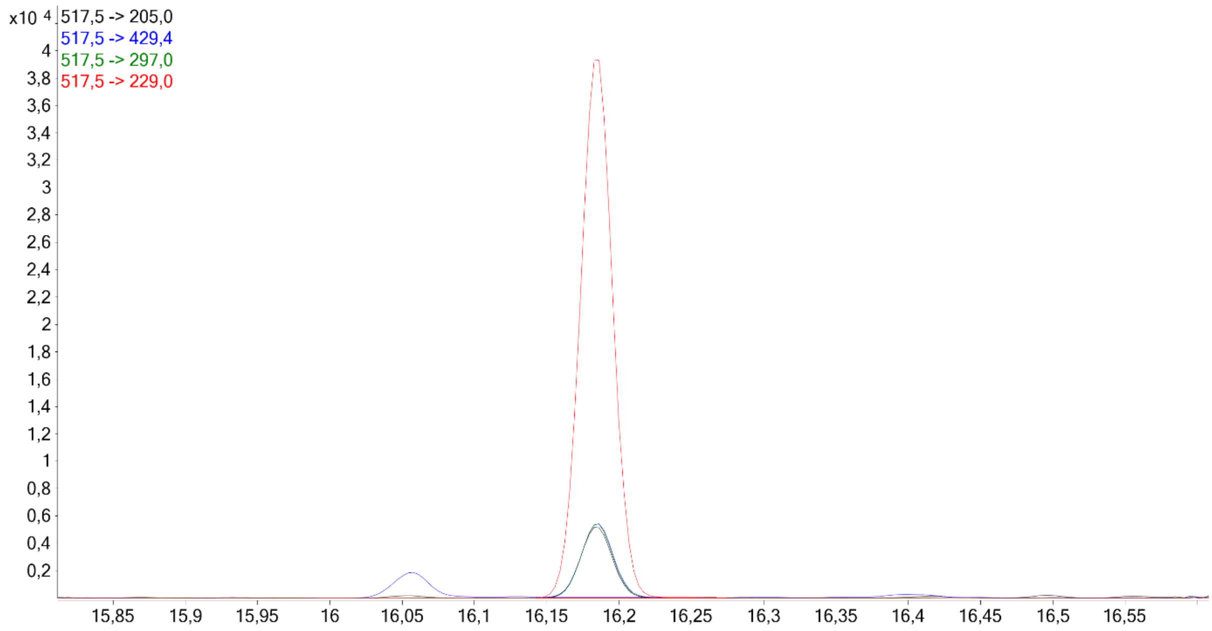

Figure S3-3: GC-QQQ-MS chromatogram of 6-OH-metandienone (M2, **13**)

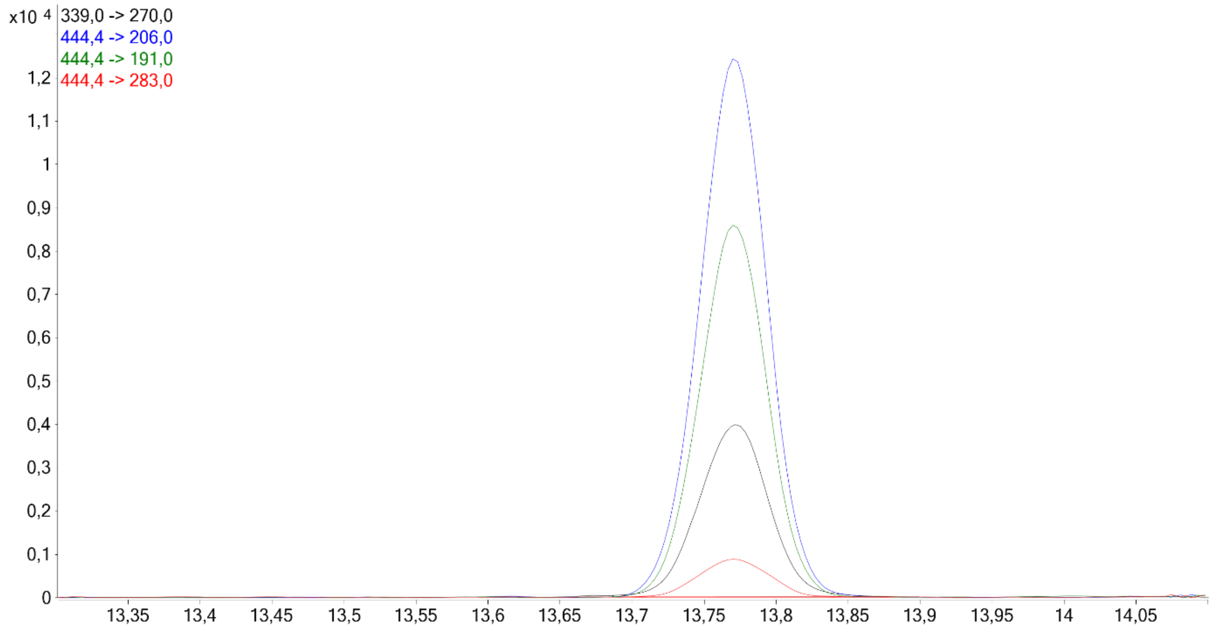

Figure S3-4: GC-QQQ-MS chromatogram of epimetandienone (M3, **14**)

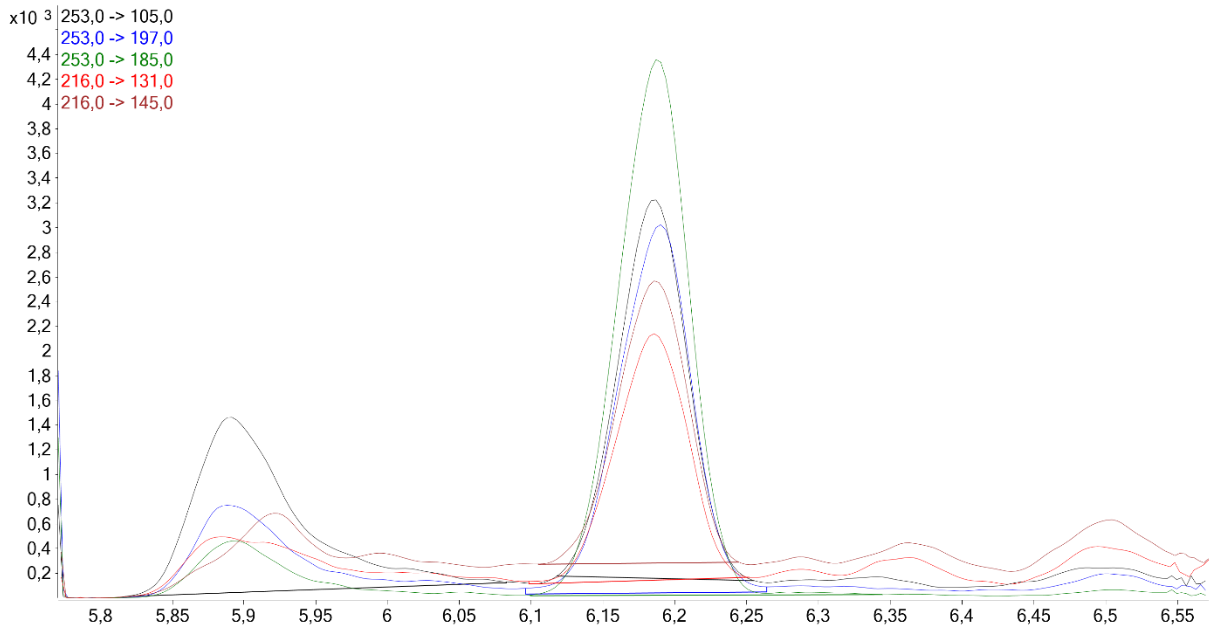

Figure S3-5: GC-QQQ-MS chromatogram of normetendiol (M4, **16**)

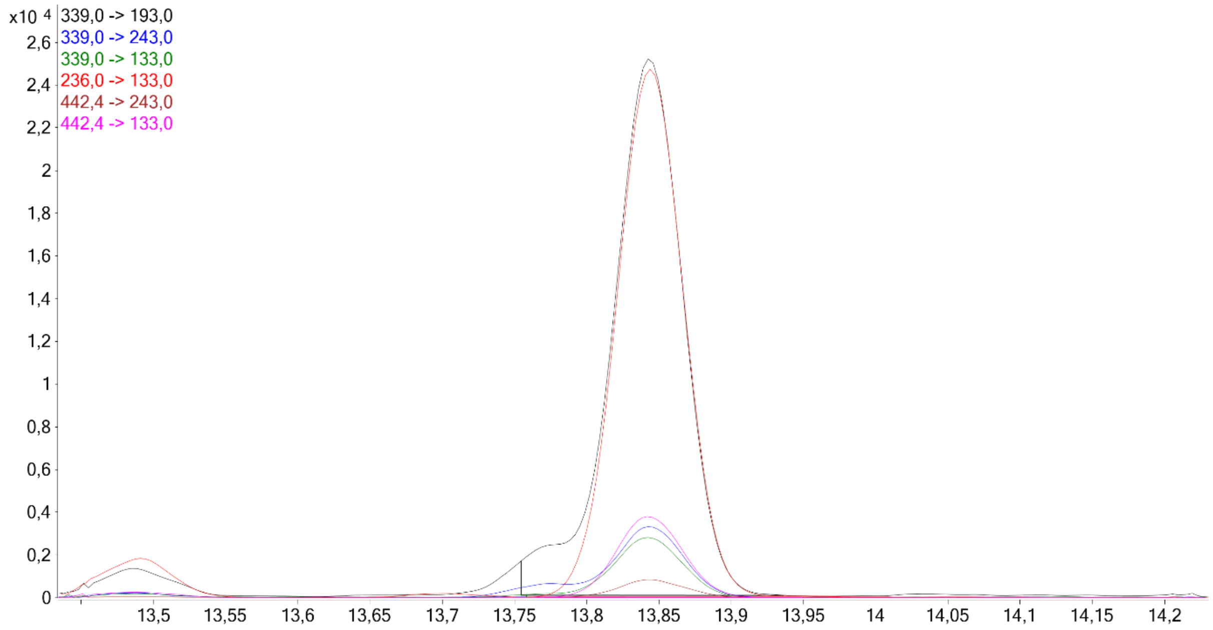

Figure S3-6: GC-QQQ-MS chromatogram of 17β-hydroxymethyl-17α-methyl-18-nor-androst-1,4,13-trien-3-one (M6, **17**)

Post administration urine of methyltestosterone, collected 38 h after ingestion

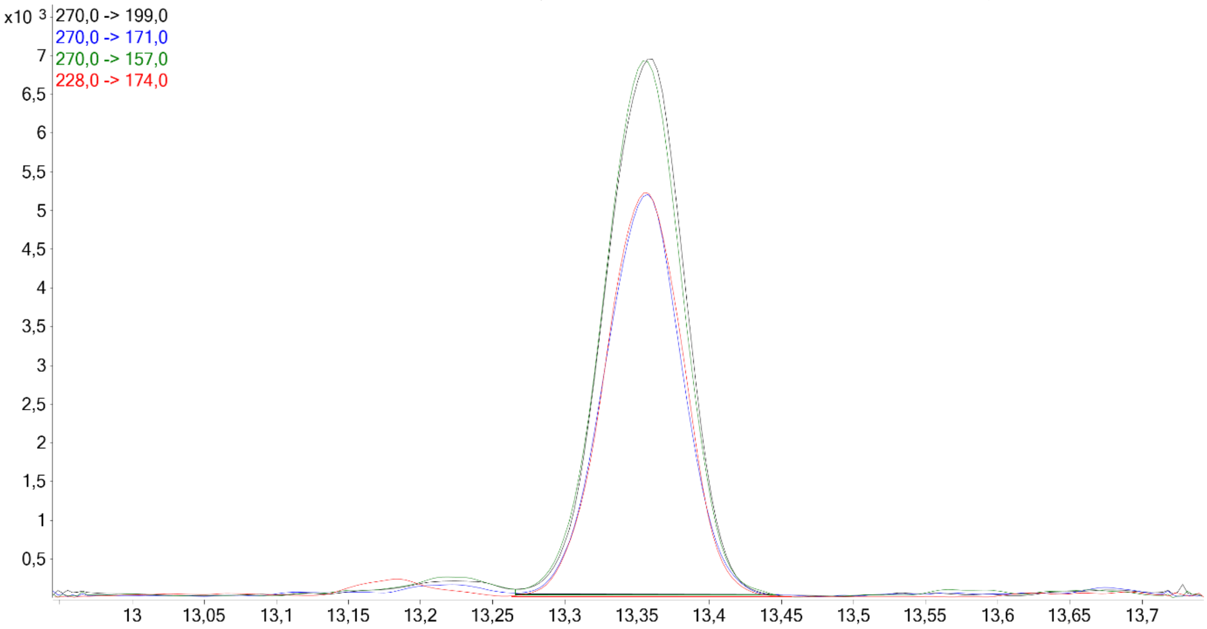

Figure S3-7: GC-QQQ-MS chromatogram of 3α5β-tetrahydromethyltestosterone (M1, **20**)

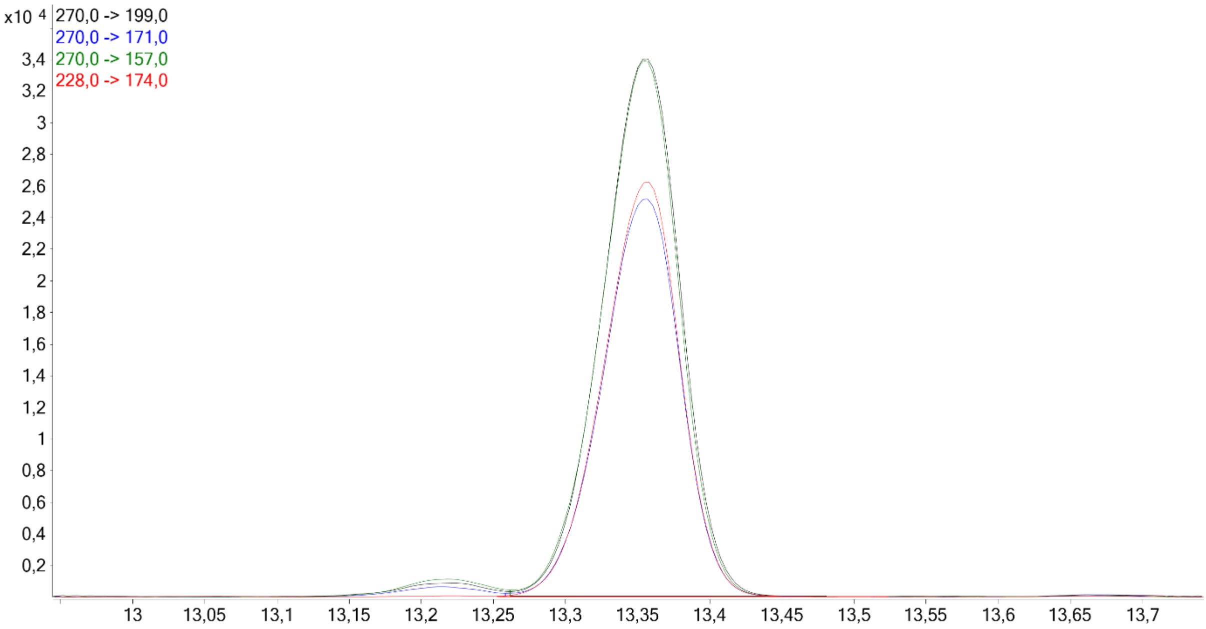

Figure S3-8: GC-QQQ-MS chromatogram of 3α5α-tetrahydromethyltestosterone (M2, **19**)
