# Supplementary material for: New Insights into the Metabolism of Methyltestosterone and Metandienone: Detection of Novel A-Ring Reduced Metabolites
Source: Molecules. 2021 Mar 3;26(5):1354. doi: 10.3390/molecules26051354 (PMC7961831; doi:10.3390/molecules26051354)
Supplement: Supplementary file 1 [file molecules-26-01354-s001.zip › molecules-1132802-final-SM/210202_Molecules_20OHTHMT_Supplement4.pdf]

molecules

MDPI

Supplement S4: Detailed amounts of reactants and solvents in the synthesis of 17-hydroxymethyl-17-methyl-18-nor-5-androst-13-en-3-ols

Table S4-1: Reactants and solvents used in the methenylation of Etiocholanolone (**5**) or androsterone (**5a**)

	**6**	**6a**
Reagent		
Nysted (20 %)	14.2 mL	13.4 mL
THF	7.0 mL	7.0 mL
$TiCl_4$	360 µL	330 µL
Precursor Steroid	**5**	**5a**
Amount of Steroid	43.0 mg	394 mg
HCl (2 M)	15 mL	25 mL
Ice-cold water	15 mL	25 mL
Diethyl ether	60 mL	100 mL

Table S4-2: Reactants and solvents used for epoxidation

	**7**	**7a**
Reagent		
Precursor Steroid	**6**	**6a**
DCM	5 mL	20 mL
$KHCO_3$	44.5 mg	440 mg
m-CPBA	31.0 mg	370 mg
H_2O	10 mL	20 mL
DCM	20 mL	50 mL
